# Di-μ-oxido-bis­[(1,4,8,11-tetra­aza­cyclo­tetra­decane-κ^4^ *N*,*N*′,*N*′′,*N*′′′)dimangan­ese(III,IV)] bis­(tetra­phenyl­borate) chloride acetonitrile disolvate

**DOI:** 10.1107/S1600536811019829

**Published:** 2011-06-04

**Authors:** Marilyn M. Olmstead, David W. Boyce, Lauren E. Bria

**Affiliations:** a Department of Chemistry, University of California, One Shields Avenue, Davis, CA 95616, USA

## Abstract

The title compound, [Mn_2_O_2_(C_10_H_24_N_4_)_2_](C_24_H_20_B)_2_Cl·2CH_3_CN, is a mixed-valent Mn^III^/Mn^IV^ oxide-bridged mangan­ese dimer with one chloride and two tetra­phenyl­borate counter-anions. There are two non-coordinated mol­ecules of acetonitrile in the formula unit. A center of inversion is present between the two metal atoms, and, consequently, there is no distinction between Mn^III^ and Mn^IV^ metal centers. In the Mn_2_O_2_ core, the Mn—O distances are 1.817 (3) and 1.821 (3) Å. The cyclam ligand is in the *cis* configuration. The chloride counter-anion resides on a center of symmetry, whereas the tetra­phenyl­borate counter-anion is in a general position. The cyclam ligand is hydrogen bonded to the acetonitrile as well as to the chloride anion. One of the phenyl rings of the anion and the acetonitrile solvent molecule are each disordered over two sets of sites.

## Related literature

For structures of different salts containing the disordered mixed-valent {[(cyclam)MnO]_2_}^3+^ cation, see: Goodson *et al.* (1990 ▶); Lu *et al.* (2001 ▶). For structures of the non-disordered Mn^III^—Mn^IV^O_2_ core, see: Brewer *et al.* (1989 ▶); Levaton & Olmstead (2010 ▶). For cyclam configurations, see: Bosnich *et al.* (1965 ▶).
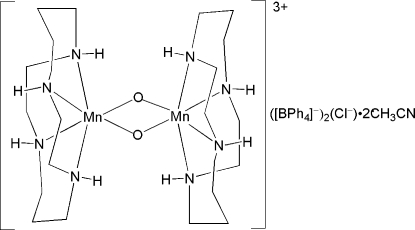


## Experimental

### 

#### Crystal data

[Mn_2_O_2_(C_10_H_24_N_4_)_2_](C_24_H_20_B)_2_Cl·2C_2_H_3_N
*M* *_r_* = 1298.52
Triclinic, 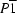

*a* = 11.437 (2) Å
*b* = 11.713 (2) Å
*c* = 13.967 (3) Å
α = 104.136 (3)°
β = 97.697 (3)°
γ = 107.627 (3)°
*V* = 1684.9 (6) Å^3^
*Z* = 1
Mo *K*α radiation
μ = 0.47 mm^−1^
*T* = 90 K
0.15 × 0.11 × 0.08 mm

#### Data collection

Bruker SMART APEXII diffractometer
Absorption correction: multi-scan (*SADABS*; Sheldrick, 1996 ▶) *T*_min_ = 0.952, *T*_max_ = 0.972
17931 measured reflections
6092 independent reflections
4083 reflections with *I* > 2σ(*I*)
*R*_int_ = 0.064

#### Refinement

*R*[*F* ^2^ > 2σ(*F* ^2^)] = 0.061
*wR*(*F* ^2^) = 0.173
*S* = 1.03
6092 reflections
433 parameters
258 restraints
H-atom parameters constrained
Δρ_max_ = 0.54 e Å^−3^
Δρ_min_ = −0.81 e Å^−3^

### 

Data collection: *APEX2* (Bruker, 2007 ▶); cell refinement: *SAINT* (Bruker, 2007 ▶); data reduction: *SAINT*; program(s) used to solve structure: *SHELXS97* (Sheldrick, 2008 ▶); program(s) used to refine structure: *SHELXL97* (Sheldrick, 2008 ▶); molecular graphics: *XP* in *SHELXTL* (Sheldrick, 2008 ▶); software used to prepare material for publication: *SHELXL97*.

## Supplementary Material

Crystal structure: contains datablock(s) global, I. DOI: 10.1107/S1600536811019829/hg5039sup1.cif

Structure factors: contains datablock(s) I. DOI: 10.1107/S1600536811019829/hg5039Isup2.hkl

Additional supplementary materials:  crystallographic information; 3D view; checkCIF report

## Figures and Tables

**Table 1:** Selected bond lengths (Å)

Mn1—O1	1.817 (3)
Mn1—O1^i^	1.821 (3)
Mn1—N1	2.187 (5)
Mn1—N2	2.092 (3)
Mn1—N3	2.178 (3)
Mn1—N4	2.116 (3)

Symmetry code: (i) 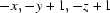.

**Table 2:** Hydrogen-bond geometry (Å, °)

*D*—H⋯*A*	*D*—H	H⋯*A*	*D*⋯*A*	*D*—H⋯*A*
N1—H1⋯N5	0.93	2.42	3.313 (11)	160
N1—H1⋯N5*B*	0.93	1.98	2.832 (10)	151
N2—H2⋯Cl1	0.93	2.37	3.289 (4)	169
N3—H3⋯N5^i^	0.93	2.22	3.034 (9)	146
N3—H3⋯N5*B*^i^	0.93	2.23	3.120 (9)	160
N4—H4⋯Cl1	0.93	2.42	3.330 (4)	168

Symmetry code: (i) 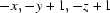.

**Fig. 1.**
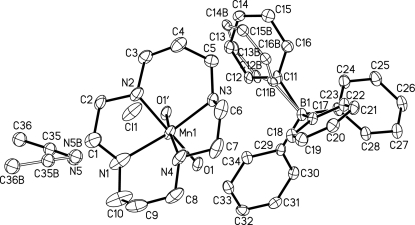
The asymmetric unit of the title complex with displacement parameters drawn at the 30% probability level. Disordered components of the acetonitrile and [BPh4]- group are shown.

**Fig. 2.**
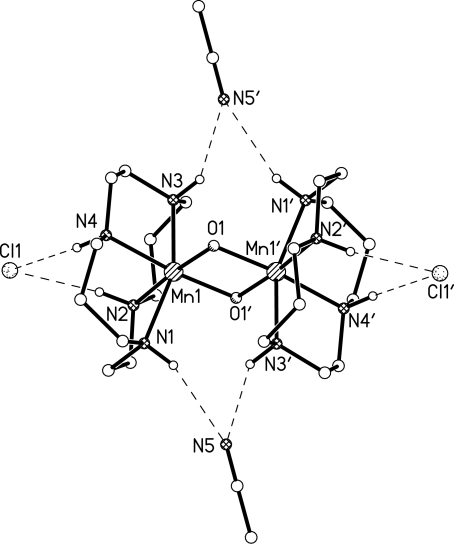
The centrosymmetric dimer of the title cation is shown with H-bonds involving the N—H groups, acetonitriles and chlorides depicted with dashed lines.

## supplementary crystallographic information

### Comment

In the title compound (Fig. 1), the Mn^III^—Mn^IV^ centrosymmetric, dinuclear cation is bridged by two oxo ligands. Each manganese atom also binds to a tetradentate cyclam (cyclam = 1,4,8,11-tetraazacyclotetradecane) to achieve a distorted octahedral coordination environment. Due to the center of inversion, there is no distinction between the two different oxidation states in the structure, and it is disordered mixed valent. The dinuclear cation bears a +3 charge, and the charge is balanced by a chloride anion and two tetraphenylborate anions. The chloride resides on a center of symmetry. The configuration of the cyclam ligand is *cis*-V, in which the N—H bonds alternate above and below the N_4_ plane (Bosnich *et al.*, 1965).

The structures of similar disordered mixed valent (III/IV) µ-oxo bridged manganese cyclam complexes are reported in Lu, *et al.*, 2001, with two perchlorate and one nitrate anions; Goodson, *et al.*, 1990, with dithionate and thiosulfate anions, and a second structure with three bromides. In the title compound, the Mn_2_O_2_ core has Mn—O distances of 1.817 (3) and 1.821 (3) Å. All of the above referenced structures show similar coordination geometry, and the mean Mn—O bond distances in the Mn_2_O_2_ core for these complexes is 1.824 Å. A trifluoromethanesulfonate salt (Brewer, *et al.*, 1989) crystallized with discrete Mn^III^ and Mn^IV^ metal centers, and these mean Mn—O distances are characteristic of the oxidation states of Mn: Mn^III^—O, 1.861 Å and Mn^IV^—O, 1.788 Å. Related values were also seen in the structure of a large Mn_12_ cluster with three units of Mn_2_O_2_ core geometry. Average values with average deviations from the mean were Mn^III^—O, 1.879 (7) Å and Mn^IV^—O, 1.787 (6) Å (Levaton & Olmstead, 2010). Thus, the disordered distances agree well with the average III/IV values.

In the structure of the title compound, all of the available hydrogen atoms bonded to the N atoms in the cyclam ligand participate in hydrogen bonding (Fig. 2 and Table 2). The hydrogen atoms bonded to N atoms N2 and N4 are hydrogen bonded to chloride atom. The hydrogen atoms on N atoms N1 and N3 hydrogen bond to the acetonitrile N atoms.

### Experimental

The title compound was obtained while attempting to prepare an azido derivative. To a stirred solution of 1,4,8,11-tetraazacyclotetradecane (cyclam), (200 mg, 1 mmol) in 5 ml of methanol was added a solution of MnCl_2_^.^4H_2_O (200 mg, 1 mmol) in 25 ml of methanol. Over the one hour course of the addition, the reaction color progressed from red to dark green. After stirring for 1 hr an excess of NaN_3_ (200 mg, 2.5 mmol) was added to the reaction mixture in a solution of 9 ml me thanol and 1 ml H_2_O. No color change was observed upon addition of the azide. After 20 min of stirring NaBPh_4_ (350 mg, 1 mmol) was added. Addition of the tetraphenylborate salt caused a precipitate to form and the solution turned red-brown. Subsequently, 18 ml of a 5:1 acetonitrile:water and 10 ml of 0.1 *M* HClO_4_ were added. The orange-brown solid was collected by filtration. Dichroic, red-green crystals of the product were obtained by slow evaporation of a toluene solution.

### Refinement

The C-bound and N-bound H atoms were positioned geometrically with C—H = 0.95—0.98 Å, N—H = 0.88 Å, and allowed to ride on their parent atoms with *U*_iso_(H) = 1.2—1.5 *U*_eq_(C). One of the BPh_4_^-^ phenyl rings was disordered and was refined in two parts with a rigid group refinement. The acetonitrile molecule was disordered in two parts, each assigned 50% occupancy. Atoms of the disordered acetonitriles were kept isotropic. The atoms of the disordered BPh_4_^-^ phenyl group were allowed to refine with anisotropic thermal parameters and a similarity restraint (SIMU) of 0.008. The cation displayed large thermal motion and thermal ellipsoids for these atoms were refined with an ISOR 0.01 restraint. An attempt was made to solve and refine the structure in the non-centrosymmetric space group P1 but atoms of the cation became N.P.D.

### Crystal data

**Table:**

[Mn_2_O_2_(C_10_H_24_N_4_)_2_](C_24_H_20_B)_2_Cl·2C_2_H_3_N	*Z* = 1
*M**_r_* = 1298.52	*F*(000) = 689
Triclinic, *P*1	*D*_x_ = 1.280 Mg m^−^^3^
Hall symbol: -P 1	Mo *K*α radiation, λ = 0.71073 Å
*a* = 11.437 (2) Å	Cell parameters from 3568 reflections
*b* = 11.713 (2) Å	θ = 2.8–24.6°
*c* = 13.967 (3) Å	µ = 0.47 mm^−^^1^
α = 104.136 (3)°	*T* = 90 K
β = 97.697 (3)°	Block, red
γ = 107.627 (3)°	0.15 × 0.11 × 0.08 mm
*V* = 1684.9 (6) Å^3^	

### Data collection

**Table:**

Bruker SMART APEXII diffractometer	6092 independent reflections
Radiation source: fine-focus sealed tube	4083 reflections with *I* > 2σ(*I*)
graphite	*R*_int_ = 0.064
Detector resolution: 8.3 pixels mm^-1^	θ_max_ = 25.3°, θ_min_ = 2.8°
ω scans	*h* = −13→13
Absorption correction: multi-scan (*SADABS*; Sheldrick, 1996)	*k* = −14→14
*T*_min_ = 0.952, *T*_max_ = 0.972	*l* = −16→16
17931 measured reflections	

### Refinement

**Table:**

Refinement on *F*^2^	Primary atom site location: structure-invariant direct methods
Least-squares matrix: full	Secondary atom site location: difference Fourier map
*R*[*F*^2^ > 2σ(*F*^2^)] = 0.061	Hydrogen site location: inferred from neighbouring sites
*wR*(*F*^2^) = 0.173	H-atom parameters constrained
*S* = 1.03	*w* = 1/[σ^2^(*F*_o_^2^) + (0.080*P*)^2^ + 1.3759*P*] where *P* = (*F*_o_^2^ + 2*F*_c_^2^)/3
6092 reflections	(Δ/σ)_max_ = 0.007
433 parameters	Δρ_max_ = 0.54 e Å^−^^3^
258 restraints	Δρ_min_ = −0.81 e Å^−^^3^

### Special details

**Table:**

Experimental. Crystals were dichroic.
Geometry. All e.s.d.'s (except the e.s.d. in the dihedral angle between two l.s. planes) are estimated using the full covariance matrix. The cell e.s.d.'s are taken into account individually in the estimation of e.s.d.'s in distances, angles and torsion angles; correlations between e.s.d.'s in cell parameters are only used when they are defined by crystal symmetry. An approximate (isotropic) treatment of cell e.s.d.'s is used for estimating e.s.d.'s involving l.s. planes.
Refinement. Refinement of *F*^2^ against ALL reflections. The weighted *R*-factor *wR* and goodness of fit *S* are based on *F*^2^, conventional *R*-factors *R* are based on *F*, with *F* set to zero for negative *F*^2^. The threshold expression of *F*^2^ > σ(*F*^2^) is used only for calculating *R*-factors(gt) *etc*. and is not relevant to the choice of reflections for refinement. *R*-factors based on *F*^2^ are statistically about twice as large as those based on *F*, and *R*- factors based on ALL data will be even larger.

### Fractional atomic coordinates and isotropic or equivalent isotropic displacement parameters (Å^2^)

**Table:**

	*x*	*y*	*z*	*U*_iso_*/*U*_eq_	Occ. (<1)
Cl1	0.5000	0.5000	0.5000	0.0774 (7)	
Mn1	0.11925 (5)	0.50038 (5)	0.50106 (5)	0.0434 (2)	
O1	−0.0251 (3)	0.4058 (2)	0.5257 (2)	0.0480 (8)	
N1	0.2187 (3)	0.6175 (4)	0.6555 (4)	0.0738 (14)	
H1	0.1742	0.6709	0.6738	0.089*	
N2	0.2773 (3)	0.6205 (3)	0.4709 (3)	0.0509 (10)	
H2	0.3380	0.5826	0.4695	0.061*	
N3	0.0891 (3)	0.3830 (3)	0.3463 (2)	0.0357 (7)	
H3	0.0049	0.3316	0.3296	0.043*	
N4	0.2090 (3)	0.3743 (3)	0.5289 (3)	0.0579 (11)	
H4	0.2942	0.4107	0.5312	0.069*	
C1	0.3437 (4)	0.7009 (5)	0.6530 (4)	0.0686 (15)	
H1B	0.4048	0.6565	0.6528	0.082*	
H1C	0.3751	0.7765	0.7130	0.082*	
C2	0.3272 (5)	0.7373 (4)	0.5578 (4)	0.0658 (15)	
H2A	0.2677	0.7837	0.5590	0.079*	
H2B	0.4090	0.7923	0.5518	0.079*	
C3	0.2571 (4)	0.6510 (4)	0.3745 (3)	0.0479 (11)	
H3A	0.3327	0.7201	0.3744	0.058*	
H3B	0.1850	0.6811	0.3705	0.058*	
C4	0.2318 (4)	0.5416 (4)	0.2834 (4)	0.0547 (12)	
H4A	0.2972	0.5034	0.2925	0.066*	
H4B	0.2384	0.5720	0.2234	0.066*	
C5	0.1037 (4)	0.4420 (4)	0.2635 (3)	0.0538 (12)	
H5A	0.0381	0.4802	0.2554	0.065*	
H5B	0.0903	0.3760	0.1991	0.065*	
C6	0.1589 (4)	0.2975 (4)	0.3453 (4)	0.0594 (13)	
H6A	0.2471	0.3397	0.3428	0.071*	
H6B	0.1202	0.2226	0.2849	0.071*	
C7	0.1555 (4)	0.2593 (4)	0.4390 (4)	0.0676 (15)	
H7A	0.2054	0.2038	0.4417	0.081*	
H7B	0.0676	0.2123	0.4393	0.081*	
C8	0.1982 (4)	0.3418 (5)	0.6219 (4)	0.0695 (16)	
H8A	0.2278	0.2704	0.6209	0.083*	
H8B	0.1086	0.3145	0.6256	0.083*	
C9	0.2735 (5)	0.4502 (5)	0.7142 (4)	0.0677 (15)	
H9A	0.3608	0.4829	0.7057	0.081*	
H9B	0.2770	0.4181	0.7735	0.081*	
C10	0.2253 (6)	0.5587 (6)	0.7381 (6)	0.098 (2)	
H10A	0.1403	0.5281	0.7515	0.117*	
H10B	0.2812	0.6235	0.8006	0.117*	
B1	0.2591 (4)	−0.1045 (4)	0.1854 (3)	0.0326 (9)	
C11	0.3193 (5)	0.0254 (4)	0.1481 (3)	0.0315 (16)	0.542 (4)
C12	0.3289 (5)	0.1438 (5)	0.2064 (3)	0.0257 (15)	0.542 (4)
H12	0.3015	0.1535	0.2682	0.031*	0.542 (4)
C13	0.3786 (5)	0.2481 (4)	0.1743 (4)	0.0308 (17)	0.542 (4)
H13	0.3852	0.3291	0.2141	0.037*	0.542 (4)
C14	0.4187 (5)	0.2339 (4)	0.0838 (4)	0.0334 (15)	0.542 (4)
H14	0.4527	0.3051	0.0618	0.040*	0.542 (4)
C15	0.4090 (4)	0.1154 (4)	0.0255 (3)	0.0401 (16)	0.542 (4)
H15	0.4364	0.1057	−0.0363	0.048*	0.542 (4)
C16	0.3593 (5)	0.0112 (3)	0.0577 (3)	0.0356 (15)	0.542 (4)
H16	0.3527	−0.0698	0.0178	0.043*	0.542 (4)
C11B	0.3432 (5)	0.0282 (5)	0.1656 (4)	0.0263 (17)	0.458 (4)
C12B	0.2979 (5)	0.1267 (7)	0.1898 (5)	0.0306 (19)	0.458 (4)
H12B	0.2276	0.1178	0.2203	0.037*	0.458 (4)
C13B	0.3555 (6)	0.2381 (5)	0.1693 (5)	0.0330 (19)	0.458 (4)
H13B	0.3245	0.3054	0.1858	0.040*	0.458 (4)
C14B	0.4583 (5)	0.2511 (4)	0.1246 (4)	0.0325 (18)	0.458 (4)
H14B	0.4977	0.3273	0.1106	0.039*	0.458 (4)
C15B	0.5037 (5)	0.1527 (5)	0.1004 (4)	0.0371 (17)	0.458 (4)
H15B	0.5740	0.1615	0.0698	0.045*	0.458 (4)
C16B	0.4461 (5)	0.0412 (4)	0.1209 (4)	0.0309 (16)	0.458 (4)
H16B	0.4771	−0.0261	0.1043	0.037*	0.458 (4)
C17	0.3410 (3)	−0.1988 (3)	0.1658 (3)	0.0321 (8)	
C18	0.4455 (4)	−0.1898 (4)	0.2359 (3)	0.0385 (9)	
H18	0.4718	−0.1249	0.2987	0.046*	
C19	0.5126 (4)	−0.2704 (4)	0.2186 (3)	0.0434 (10)	
H19	0.5823	−0.2602	0.2694	0.052*	
C20	0.4795 (4)	−0.3655 (4)	0.1283 (3)	0.0413 (9)	
H20	0.5247	−0.4216	0.1163	0.050*	
C21	0.3785 (4)	−0.3764 (3)	0.0563 (3)	0.0387 (9)	
H21	0.3543	−0.4401	−0.0071	0.046*	
C22	0.3121 (3)	−0.2956 (3)	0.0752 (3)	0.0350 (9)	
H22	0.2427	−0.3065	0.0238	0.042*	
C23	0.1172 (3)	−0.1789 (3)	0.1131 (3)	0.0338 (8)	
C24	0.0576 (4)	−0.1332 (4)	0.0454 (3)	0.0405 (9)	
H24	0.1015	−0.0532	0.0393	0.049*	
C25	−0.0642 (4)	−0.2001 (4)	−0.0140 (3)	0.0444 (10)	
H25	−0.1010	−0.1655	−0.0595	0.053*	
C26	−0.1310 (4)	−0.3161 (4)	−0.0067 (3)	0.0428 (10)	
H26	−0.2145	−0.3611	−0.0458	0.051*	
C27	−0.0750 (4)	−0.3662 (4)	0.0581 (3)	0.0403 (9)	
H27	−0.1190	−0.4469	0.0626	0.048*	
C28	0.0459 (3)	−0.2981 (3)	0.1165 (3)	0.0357 (9)	
H28	0.0822	−0.3340	0.1611	0.043*	
C29	0.2486 (3)	−0.0639 (3)	0.3049 (3)	0.0291 (8)	
C30	0.1384 (3)	−0.0995 (3)	0.3392 (3)	0.0341 (8)	
H30	0.0621	−0.1500	0.2908	0.041*	
C31	0.1344 (4)	−0.0647 (3)	0.4414 (3)	0.0393 (9)	
H31	0.0564	−0.0909	0.4608	0.047*	
C32	0.2429 (4)	0.0075 (3)	0.5140 (3)	0.0380 (9)	
H32	0.2413	0.0289	0.5838	0.046*	
C33	0.3534 (4)	0.0479 (3)	0.4835 (3)	0.0373 (9)	
H33	0.4290	0.0997	0.5323	0.045*	
C34	0.3548 (3)	0.0130 (3)	0.3813 (3)	0.0353 (9)	
H34	0.4325	0.0432	0.3624	0.042*	
N5	0.1329 (9)	0.8609 (9)	0.7468 (7)	0.053 (2)*	0.50
C35	0.1548 (8)	0.9672 (8)	0.7751 (6)	0.0402 (18)*	0.50
C36	0.1803 (8)	1.0976 (8)	0.8123 (7)	0.050 (2)*	0.50
H36A	0.1058	1.1123	0.8322	0.076*	0.50
H36B	0.2014	1.1365	0.7593	0.076*	0.50
H36C	0.2514	1.1346	0.8713	0.076*	0.50
N5B	0.1679 (8)	0.8389 (8)	0.7379 (6)	0.047 (2)*	0.50
C35B	0.2034 (8)	0.9323 (8)	0.7992 (6)	0.0432 (19)*	0.50
C36B	0.2519 (9)	1.0513 (9)	0.8797 (7)	0.059 (2)*	0.50
H36D	0.2796	1.0372	0.9440	0.088*	0.50
H36E	0.1856	1.0877	0.8854	0.088*	0.50
H36F	0.3234	1.1091	0.8641	0.088*	0.50

### Atomic displacement parameters (Å^2^)

**Table:**

	*U*^11^	*U*^22^	*U*^33^	*U*^12^	*U*^13^	*U*^23^
Cl1	0.0257 (8)	0.0762 (12)	0.1513 (19)	0.0152 (8)	0.0083 (9)	0.0805 (13)
Mn1	0.0283 (3)	0.0300 (3)	0.0637 (4)	−0.0002 (2)	−0.0153 (3)	0.0263 (3)
O1	0.0452 (16)	0.0273 (13)	0.0557 (17)	−0.0017 (12)	−0.0218 (13)	0.0209 (13)
N1	0.034 (2)	0.083 (3)	0.134 (4)	0.026 (2)	0.020 (2)	0.079 (3)
N2	0.0361 (18)	0.0423 (19)	0.070 (2)	0.0029 (15)	−0.0116 (17)	0.0352 (18)
N3	0.0269 (16)	0.0314 (16)	0.0460 (19)	0.0098 (13)	0.0047 (14)	0.0092 (14)
N4	0.0307 (18)	0.045 (2)	0.097 (3)	0.0030 (15)	−0.0096 (18)	0.045 (2)
C1	0.032 (2)	0.086 (4)	0.081 (3)	0.002 (2)	−0.006 (2)	0.047 (3)
C2	0.052 (3)	0.049 (3)	0.065 (3)	−0.020 (2)	−0.023 (2)	0.030 (2)
C3	0.043 (2)	0.041 (2)	0.065 (3)	0.0157 (19)	0.001 (2)	0.029 (2)
C4	0.051 (3)	0.047 (2)	0.078 (3)	0.020 (2)	0.034 (2)	0.026 (2)
C5	0.061 (3)	0.054 (3)	0.040 (2)	0.010 (2)	0.019 (2)	0.013 (2)
C6	0.041 (2)	0.039 (2)	0.094 (4)	0.019 (2)	0.011 (2)	0.009 (2)
C7	0.044 (3)	0.032 (2)	0.120 (4)	0.011 (2)	−0.013 (3)	0.030 (3)
C8	0.037 (2)	0.070 (3)	0.115 (4)	0.012 (2)	−0.001 (3)	0.070 (3)
C9	0.068 (3)	0.092 (4)	0.089 (4)	0.053 (3)	0.035 (3)	0.068 (3)
C10	0.099 (4)	0.115 (4)	0.154 (5)	0.072 (4)	0.082 (4)	0.100 (4)
B1	0.033 (2)	0.026 (2)	0.032 (2)	0.0078 (18)	0.0034 (18)	0.0023 (18)
C11	0.020 (3)	0.033 (3)	0.030 (3)	0.006 (2)	−0.005 (2)	0.001 (2)
C12	0.014 (3)	0.028 (3)	0.027 (3)	0.005 (2)	−0.007 (2)	0.006 (2)
C13	0.021 (3)	0.030 (3)	0.036 (3)	0.007 (2)	−0.005 (3)	0.007 (3)
C14	0.025 (3)	0.039 (3)	0.038 (3)	0.010 (2)	0.000 (3)	0.022 (2)
C15	0.035 (3)	0.046 (3)	0.042 (3)	0.018 (3)	0.007 (3)	0.015 (3)
C16	0.034 (3)	0.037 (3)	0.034 (3)	0.013 (2)	0.006 (2)	0.010 (2)
C11B	0.028 (3)	0.028 (3)	0.021 (3)	0.012 (2)	−0.006 (3)	0.009 (2)
C12B	0.023 (3)	0.030 (3)	0.027 (3)	0.006 (3)	−0.011 (3)	0.002 (3)
C13B	0.024 (3)	0.032 (3)	0.034 (3)	0.010 (3)	−0.010 (3)	0.005 (3)
C14B	0.033 (3)	0.033 (3)	0.024 (3)	0.005 (3)	−0.006 (3)	0.009 (3)
C15B	0.037 (3)	0.039 (3)	0.032 (3)	0.010 (3)	0.007 (3)	0.010 (3)
C16B	0.033 (3)	0.032 (3)	0.027 (3)	0.014 (3)	0.003 (3)	0.006 (3)
C17	0.0302 (19)	0.0253 (18)	0.035 (2)	0.0055 (15)	0.0050 (16)	0.0053 (15)
C18	0.034 (2)	0.036 (2)	0.038 (2)	0.0101 (17)	0.0039 (17)	0.0038 (17)
C19	0.038 (2)	0.044 (2)	0.047 (2)	0.0149 (19)	0.0024 (18)	0.014 (2)
C20	0.043 (2)	0.035 (2)	0.049 (2)	0.0177 (18)	0.0129 (19)	0.0111 (19)
C21	0.042 (2)	0.0259 (19)	0.043 (2)	0.0102 (17)	0.0102 (18)	0.0023 (17)
C22	0.032 (2)	0.0282 (19)	0.037 (2)	0.0063 (16)	0.0026 (16)	0.0037 (16)
C23	0.038 (2)	0.0288 (19)	0.0294 (19)	0.0145 (17)	0.0036 (16)	−0.0010 (15)
C24	0.045 (2)	0.032 (2)	0.038 (2)	0.0120 (18)	−0.0007 (18)	0.0047 (17)
C25	0.045 (2)	0.042 (2)	0.039 (2)	0.018 (2)	−0.0056 (18)	0.0048 (18)
C26	0.036 (2)	0.039 (2)	0.038 (2)	0.0102 (18)	−0.0070 (18)	−0.0029 (18)
C27	0.037 (2)	0.032 (2)	0.042 (2)	0.0108 (17)	−0.0014 (18)	−0.0001 (17)
C28	0.032 (2)	0.032 (2)	0.035 (2)	0.0112 (16)	−0.0029 (16)	0.0024 (16)
C29	0.0282 (19)	0.0228 (17)	0.034 (2)	0.0090 (15)	0.0036 (15)	0.0065 (15)
C30	0.031 (2)	0.0270 (18)	0.040 (2)	0.0086 (16)	0.0029 (16)	0.0073 (16)
C31	0.042 (2)	0.033 (2)	0.051 (2)	0.0133 (18)	0.0187 (19)	0.0204 (18)
C32	0.056 (3)	0.035 (2)	0.031 (2)	0.0222 (19)	0.0103 (18)	0.0146 (17)
C33	0.037 (2)	0.036 (2)	0.033 (2)	0.0134 (17)	−0.0016 (17)	0.0041 (17)
C34	0.032 (2)	0.035 (2)	0.033 (2)	0.0094 (16)	0.0041 (16)	0.0040 (16)

### Geometric parameters (Å, °)

**Table:**

Mn1—O1	1.817 (3)	C15—C16	1.3900
Mn1—O1^i^	1.821 (3)	C15—H15	0.9500
Mn1—N1	2.187 (5)	C16—H16	0.9500
Mn1—N2	2.092 (3)	C11B—C12B	1.3900
Mn1—N3	2.178 (3)	C11B—C16B	1.3900
Mn1—N4	2.116 (3)	C12B—C13B	1.3900
Mn1—Mn1^i^	2.7211 (13)	C12B—H12B	0.9500
N1—C1	1.478 (6)	C13B—C14B	1.3900
N1—C10	1.485 (7)	C13B—H13B	0.9500
N1—H1	0.9300	C14B—C15B	1.3900
N2—C3	1.482 (5)	C14B—H14B	0.9500
N2—C2	1.484 (6)	C15B—C16B	1.3900
N2—H2	0.9300	C15B—H15B	0.9500
N3—C6	1.456 (5)	C16B—H16B	0.9500
N3—C5	1.488 (5)	C17—C18	1.399 (5)
N3—H3	0.9300	C17—C22	1.399 (5)
N4—C8	1.450 (6)	C18—C19	1.385 (5)
N4—C7	1.495 (6)	C18—H18	0.9500
N4—H4	0.9300	C19—C20	1.380 (6)
C1—C2	1.497 (6)	C19—H19	0.9500
C1—H1B	0.9900	C20—C21	1.379 (5)
C1—H1C	0.9900	C20—H20	0.9500
C2—H2A	0.9900	C21—C22	1.382 (5)
C2—H2B	0.9900	C21—H21	0.9500
C3—C4	1.492 (6)	C22—H22	0.9500
C3—H3A	0.9900	C23—C24	1.393 (5)
C3—H3B	0.9900	C23—C28	1.404 (5)
C4—C5	1.511 (6)	C24—C25	1.398 (5)
C4—H4A	0.9900	C24—H24	0.9500
C4—H4B	0.9900	C25—C26	1.379 (6)
C5—H5A	0.9900	C25—H25	0.9500
C5—H5B	0.9900	C26—C27	1.379 (5)
C6—C7	1.484 (7)	C26—H26	0.9500
C6—H6A	0.9900	C27—C28	1.387 (5)
C6—H6B	0.9900	C27—H27	0.9500
C7—H7A	0.9900	C28—H28	0.9500
C7—H7B	0.9900	C29—C30	1.391 (5)
C8—C9	1.503 (7)	C29—C34	1.395 (5)
C8—H8A	0.9900	C30—C31	1.396 (5)
C8—H8B	0.9900	C30—H30	0.9500
C9—C10	1.516 (7)	C31—C32	1.375 (5)
C9—H9A	0.9900	C31—H31	0.9500
C9—H9B	0.9900	C32—C33	1.372 (6)
C10—H10A	0.9900	C32—H32	0.9500
C10—H10B	0.9900	C33—C34	1.389 (5)
B1—C23	1.639 (5)	C33—H33	0.9500
B1—C17	1.649 (5)	C34—H34	0.9500
B1—C29	1.651 (5)	N5—C35	1.146 (11)
B1—C11B	1.672 (6)	C35—C36	1.411 (11)
B1—C11	1.705 (6)	C36—H36A	0.9800
C11—C12	1.3900	C36—H36B	0.9800
C11—C16	1.3900	C36—H36C	0.9800
C12—C13	1.3900	N5B—C35B	1.129 (11)
C12—H12	0.9500	C35B—C36B	1.454 (12)
C13—C14	1.3900	C36B—H36D	0.9800
C13—H13	0.9500	C36B—H36E	0.9800
C14—C15	1.3900	C36B—H36F	0.9800
C14—H14	0.9500		
			
O1—Mn1—O1^i^	83.18 (11)	C17—B1—C11B	106.0 (4)
O1—Mn1—N1	100.05 (14)	C29—B1—C11B	105.6 (3)
O1—Mn1—N2	174.88 (13)	C23—B1—C11	105.4 (3)
O1—Mn1—N3	95.63 (12)	C17—B1—C11	111.8 (3)
O1—Mn1—N4	90.76 (13)	C29—B1—C11	110.7 (3)
O1^i^—Mn1—N1	97.81 (13)	C12—C11—C16	120.0
O1^i^—Mn1—N2	91.73 (13)	C12—C11—B1	121.2 (3)
O1^i^—Mn1—N3	97.79 (12)	C16—C11—B1	118.8 (3)
O1^i^—Mn1—N4	173.47 (12)	C13—C12—C11	120.0
N1—Mn1—N2	80.14 (14)	C13—C12—H12	120.0
N1—Mn1—N3	159.04 (14)	C11—C12—H12	120.0
N1—Mn1—N4	85.59 (15)	C12—C13—C14	120.0
N2—Mn1—N3	85.49 (13)	C12—C13—H13	120.0
N2—Mn1—N4	94.35 (14)	C14—C13—H13	120.0
N3—Mn1—N4	80.34 (14)	C15—C14—C13	120.0
Mn1—O1—Mn1^i^	96.82 (11)	C15—C14—H14	120.0
C1—N1—C10	112.3 (4)	C13—C14—H14	120.0
C1—N1—Mn1	108.9 (3)	C14—C15—C16	120.0
C10—N1—Mn1	119.8 (4)	C14—C15—H15	120.0
C1—N1—H1	104.8	C16—C15—H15	120.0
C10—N1—H1	104.8	C15—C16—C11	120.0
Mn1—N1—H1	104.8	C15—C16—H16	120.0
C3—N2—C2	110.2 (3)	C11—C16—H16	120.0
C3—N2—Mn1	116.2 (2)	C12B—C11B—C16B	120.0
C2—N2—Mn1	107.2 (3)	C12B—C11B—B1	115.4 (4)
C3—N2—H2	107.7	C16B—C11B—B1	124.5 (4)
C2—N2—H2	107.7	C13B—C12B—C11B	120.0
Mn1—N2—H2	107.7	C13B—C12B—H12B	120.0
C6—N3—C5	112.2 (3)	C11B—C12B—H12B	120.0
C6—N3—Mn1	109.4 (3)	C12B—C13B—C14B	120.0
C5—N3—Mn1	119.7 (2)	C12B—C13B—H13B	120.0
C6—N3—H3	104.7	C14B—C13B—H13B	120.0
C5—N3—H3	104.7	C15B—C14B—C13B	120.0
Mn1—N3—H3	104.7	C15B—C14B—H14B	120.0
C8—N4—C7	110.7 (4)	C13B—C14B—H14B	120.0
C8—N4—Mn1	115.8 (3)	C14B—C15B—C16B	120.0
C7—N4—Mn1	106.5 (2)	C14B—C15B—H15B	120.0
C8—N4—H4	107.8	C16B—C15B—H15B	120.0
C7—N4—H4	107.8	C15B—C16B—C11B	120.0
Mn1—N4—H4	107.8	C15B—C16B—H16B	120.0
N1—C1—C2	106.8 (4)	C11B—C16B—H16B	120.0
N1—C1—H1B	110.4	C18—C17—C22	113.6 (3)
C2—C1—H1B	110.4	C18—C17—B1	124.2 (3)
N1—C1—H1C	110.4	C22—C17—B1	122.2 (3)
C2—C1—H1C	110.4	C19—C18—C17	123.4 (4)
H1B—C1—H1C	108.6	C19—C18—H18	118.3
N2—C2—C1	107.9 (4)	C17—C18—H18	118.3
N2—C2—H2A	110.1	C20—C19—C18	120.8 (4)
C1—C2—H2A	110.1	C20—C19—H19	119.6
N2—C2—H2B	110.1	C18—C19—H19	119.6
C1—C2—H2B	110.1	C21—C20—C19	117.7 (4)
H2A—C2—H2B	108.4	C21—C20—H20	121.1
N2—C3—C4	113.0 (3)	C19—C20—H20	121.1
N2—C3—H3A	109.0	C20—C21—C22	120.7 (4)
C4—C3—H3A	109.0	C20—C21—H21	119.7
N2—C3—H3B	109.0	C22—C21—H21	119.7
C4—C3—H3B	109.0	C21—C22—C17	123.8 (3)
H3A—C3—H3B	107.8	C21—C22—H22	118.1
C3—C4—C5	113.3 (4)	C17—C22—H22	118.1
C3—C4—H4A	108.9	C24—C23—C28	114.8 (3)
C5—C4—H4A	108.9	C24—C23—B1	125.1 (3)
C3—C4—H4B	108.9	C28—C23—B1	120.2 (3)
C5—C4—H4B	108.9	C23—C24—C25	122.8 (4)
H4A—C4—H4B	107.7	C23—C24—H24	118.6
N3—C5—C4	112.7 (4)	C25—C24—H24	118.6
N3—C5—H5A	109.1	C26—C25—C24	120.1 (4)
C4—C5—H5A	109.1	C26—C25—H25	119.9
N3—C5—H5B	109.1	C24—C25—H25	119.9
C4—C5—H5B	109.1	C25—C26—C27	119.2 (4)
H5A—C5—H5B	107.8	C25—C26—H26	120.4
N3—C6—C7	108.2 (4)	C27—C26—H26	120.4
N3—C6—H6A	110.1	C26—C27—C28	119.8 (4)
C7—C6—H6A	110.1	C26—C27—H27	120.1
N3—C6—H6B	110.1	C28—C27—H27	120.1
C7—C6—H6B	110.1	C27—C28—C23	123.4 (4)
H6A—C6—H6B	108.4	C27—C28—H28	118.3
C6—C7—N4	109.0 (3)	C23—C28—H28	118.3
C6—C7—H7A	109.9	C30—C29—C34	114.1 (3)
N4—C7—H7A	109.9	C30—C29—B1	125.2 (3)
C6—C7—H7B	109.9	C34—C29—B1	120.7 (3)
N4—C7—H7B	109.9	C29—C30—C31	123.2 (3)
H7A—C7—H7B	108.3	C29—C30—H30	118.4
N4—C8—C9	112.1 (4)	C31—C30—H30	118.4
N4—C8—H8A	109.2	C32—C31—C30	120.2 (4)
C9—C8—H8A	109.2	C32—C31—H31	119.9
N4—C8—H8B	109.2	C30—C31—H31	119.9
C9—C8—H8B	109.2	C33—C32—C31	118.7 (4)
H8A—C8—H8B	107.9	C33—C32—H32	120.7
C8—C9—C10	116.1 (5)	C31—C32—H32	120.7
C8—C9—H9A	108.3	C32—C33—C34	120.0 (4)
C10—C9—H9A	108.3	C32—C33—H33	120.0
C8—C9—H9B	108.3	C34—C33—H33	120.0
C10—C9—H9B	108.3	C33—C34—C29	123.7 (4)
H9A—C9—H9B	107.4	C33—C34—H34	118.1
N1—C10—C9	113.5 (5)	C29—C34—H34	118.1
N1—C10—H10A	108.9	N5—C35—C36	178.4 (10)
C9—C10—H10A	108.9	N5B—C35B—C36B	178.2 (11)
N1—C10—H10B	108.9	C35B—C36B—H36D	109.5
C9—C10—H10B	108.9	C35B—C36B—H36E	109.5
H10A—C10—H10B	107.7	H36D—C36B—H36E	109.5
C23—B1—C17	108.1 (3)	C35B—C36B—H36F	109.5
C23—B1—C29	109.1 (3)	H36D—C36B—H36F	109.5
C17—B1—C29	111.6 (3)	H36E—C36B—H36F	109.5
C23—B1—C11B	116.5 (4)		

Symmetry codes: (i) −*x*, −*y*+1, −*z*+1.

### Hydrogen-bond geometry (Å, °)

**Table:**

*D*—H···*A*	*D*—H	H···*A*	*D*···*A*	*D*—H···*A*
N1—H1···N5	0.93	2.42	3.313 (11)	160
N1—H1···N5B	0.93	1.98	2.832 (10)	151
N2—H2···Cl1	0.93	2.37	3.289 (4)	169
N3—H3···N5^i^	0.93	2.22	3.034 (9)	146
N3—H3···N5B^i^	0.93	2.23	3.120 (9)	160
N4—H4···Cl1	0.93	2.42	3.330 (4)	168

Symmetry codes: (i) −*x*, −*y*+1, −*z*+1.
